# Hepatitis B discrimination: global responses requiring global data

**DOI:** 10.1186/s12889-024-18918-8

**Published:** 2024-06-11

**Authors:** Catherine Freeland, Anousha Qureshi, Jack Wallace, Kenneth Kabagambe, Hailemichael Desalegn, Chris Munoz, Dee Lee, Theobald Owusu-Ansah, Danjuma Adda, Gibril Ndow, Sarra Yousif, Hala Abdalla, Omer Kheir, Thomas Tu, Chari Cohen

**Affiliations:** 1 Hepatitis B Foundation, Doylestown, PA USA; 2 Coalition to Eradicate Viral Hepatitis in Asia Pacific, Singapore, Singapore; 3 Burnet Institute, Melbourne, Australia; 4 Centre for Social Research in Health, University of New South Wales, Sydney, Australia; 5 The National Organisation for People Living with Hepatitis B (NOPLHB), Kampala, Uganda; 6 St.Paul’s Hospital Millenium Medical College, Addis Ababa, Ethiopia; 7 Yellow Warriors Society of the Philippines, Baguio, Philippines; 8 Inno Community Development Organisation, Guangzhou, China; 9 Hepatitis Foundation of Ghana, Accra, Ghana; 10 Center For Initiative and Development, Taraba, Nigeria; 11 World Hepatitis Alliance (WHA), London, UK; 12 Medical Research Council Unit, The Gambia at London School of Hygiene and Tropical Medicine, Serrekunda, Gambia; 13 Division of Digestive Diseases, Imperial College London, London, UK; 14 Public Health Unit, Population Health, Johns Hopkins Aramco Healthcare, Dhahran, Saudi Arabia; 15 Storr Liver Centre, The Westmead Institute for Medical Research, The University of Sydney at Westmead Hospital, Westmead, Australia; 16 Centre for Infectious Diseases and Microbiology, Sydney Infectious Diseases Institute, The University of Sydney at Westmead Hospital, Westmead, Australia; 17 HepBCommunity.org, Sydney, Australia

**Keywords:** Hepatitis B, Discrimination, Stigma, Liver cancer, Liver disease, Viral hepatitis

## Abstract

Over 250 million individuals live with chronic hepatitis B (CHB) infection worldwide. A significant proportion of these people often face discrimination defined as the unjust, unfair, or prejudicial treatment of a person on the grounds of their hepatitis B status. Hepatitis B related discrimination has not been widely documented in the literature. This study aims to describe the lived experience of discrimination, document its impact, and shed light on its consequences. A hepatitis B discrimination registry was launched to record self-reported discrimination associated with hepatitis B. The registry included brief demographic questions (age, gender, country of origin), discrimination-specific questions (where, when, and how discrimination occurred), and open-ended questions to detail specific experiences. The registry was distributed to hepatitis B patient/people-focused listservs, social media networks, and community-based organizations around the globe. Descriptive data were analyzed including comparative analysis by country and type of discrimination occurring along with qualitative data (open-ended responses) which were analyzed using thematic analysis techniques A total of 569 individuals responded to the survey between May 2021 and December 2023. Individuals identified as residing in the Philippines (34%; *N* = 194), Nigeria (11%; *N* = 60), Pakistan (8%; *N* = 45), India (6%, *N* = 34), Uganda (5%; *N* = 31), the United States of America (4%, *N* = 26), Ghana (3%; *N* = 15), Ethiopia (2%; *N* = 14), and other countries in smaller number with a total of 65 countries reported discrimination at least by one individual. Of these, 461 individuals shared details about their experiences of discrimination with most relating to restrictions on access to work visas, followed by in-country hepatitis B-related employment restrictions, educational-based discrimination, discrimination within the community and health facilities, and the emotional impact of hepatitis B discrimination. This is the largest primary collection of hepatitis B-associated discrimination events and highlights how hepatitis B discrimination clearly has a significant impact on individuals’ lives and limits economic opportunities regardless of physical symptoms. Such impacts likely act as barriers to diagnosis and engagement in care, so need to be addressed to achieve the global hepatitis B elimination goals. The data highlight a need for global, national responses and more systematic responses to discrimination experienced by people with hepatitis B.

## Background

Over 250 million individuals live with chronic hepatitis B (CHB) infection worldwide, most of whom have not been diagnosed and remain at risk of liver disease [1]. In 2016, the World Health Organization identified elimination goals to reduce new hepatitis B infections by 90% and hepatitis-related deaths by 65% by 2030 [2]. In May 2022, the 75th World Health Assembly convened to revise the hepatitis B elimination goals and strategies to account for disruption in services due to the COVID-19 pandemic and address disparities experienced by impacted communities [3]. These revised goals include reducing the number of annual hepatitis-related deaths to 4 per 100,000 by 2030; expanding treatment coverage for those diagnosed with hepatitis B by 80%; and reducing drug prices to treat hepatitis B by 60% [4]. While these goals are essential towards reducing hepatitis B transmission, burden, and mortality, it is equally important to understand the psychological and social consequences that often follow a hepatitis B diagnosis and their direct or indirect impact on achieving these elimination goals [5]. 

As countries work to increase testing and diagnosis, a better understanding of the lived experiences of individuals living with hepatitis B is essential. Discrimination specific to hepatitis B is defined as the unjust, unfair, or prejudicial treatment of a person on the grounds of their hepatitis B status [5]. Stigma is defined as a social process, experienced or anticipated, and is characterized by exclusion, rejection, blame, or devaluation resulting from experience, perception, or reasonable anticipation of an adverse social judgment about a person or a group [6]. Hepatitis B stigma and discrimination have been reported as a significant consequence of diagnosis for many people [5, 7–9]. Although formal data is limited, hepatitis B-related stigma and discrimination is known to have significant consequences such as denial of employment or educational opportunities, unfair treatment at work, school or home, and prohibitions on a person’s ability to emigrate to certain countries or serve in the military or police forces [5, 10–13].

So far discrimination is anecdotally reported within the global context among patient communities and advocacy groups and has significantly appeared as a key feature in many countries. Despite its significance, discrimination and stigma remains poorly reported and documented in many countries and settings. Formal structured reporting for hepatitis related stigma and discrimination are urgently needed to ensure protections of the rights of people with lived experience, prevent the consequences associated with discrimination and stigma, and improve health outcomes [14]. Our research aims to identify the settings and countries in which people with hepatitis B are most at risk of discrimination and to document the scope of this issue.

We previously identified discrimination occurring across the lifespan and described its impact on the social, emotional, and professional lives of people living with hepatitis B [5]. A major outcome from the previous study, which was qualitative in nature and collected data from key informants, was the need to identify the lived experience of discrimination from those directly impacted [5]. The goal of this current study was to build on previous learnings, to systematically document and track hepatitis B-related discrimination from individuals with direct experience, specifically in terms of the type of discrimination (employment, immigration, education) and context/setting/locations where discrimination occurs globally. We also sought to further elucidate the experiences and impacts of discrimination on those living with hepatitis B.

## Methods

In May 2021, the Hepatitis B Foundation launched an online hepatitis B discrimination registry to provide an opportunity for those experiencing discrimination to share their lived experiences and receive support if it was desired [5]. The registry’s goal is to serve as a living document to track and inform hepatitis-B-related discrimination globally.

### Data collection

The discrimination registry utilized the SurveyMonkey platform and was pilot tested and reviewed by experts including people with hepatitis B, public health researchers, and community advocates to ensure cultural competency. The survey included four brief demographic questions (age, gender, city, country of origin), eight discrimination-specific questions (where, when, and how discrimination occurred), and two open-ended questions to provide specific details regarding the discrimination experiences of those with hepatitis B. The registry was available in English language and continuously distributed to patient-focused listservs, patient support groups, social media networks, and community-based organizations around the globe. Data were collected for 30 months (between May 2021 and December 2023). All registry participation was voluntary and anonymous with participants being provided the option to include an email address if they wished to be contacted by the Hepatitis B Foundation regarding their discrimination experiences. No identifiable information was included in this analysis. The survey and its analysis are approved by the Heartland Institutional Review Board (HIRB Project No. 04202023-473).

### Data analysis

Completed survey data were extracted from the SurveyMonkey platform into Excel software and analyzed descriptively to assess the location, type/nature, and characteristics of the discrimination. Comparisons were made between the country of residence and the type of discrimination reported. Using qualitative thematic analysis techniques, two research team members double coded the open-ended responses using Nvivo software (Release 1.7.1) [15]. After reading a subsample of item responses, a codebook was used to guide the organization of the data from open-ended questions. Intercoder reliability was assessed at a kappa score of 0.80 or greater, and the study team met to resolve and discuss any discrepancies related to the qualitative analysis. All efforts were made to ensure data saturation was met for the qualitative data and that researchers have collected enough data to draw appropriate conclusions based on survey responses [16]. 

## Results

### Descriptive data

**Fig. 1:**
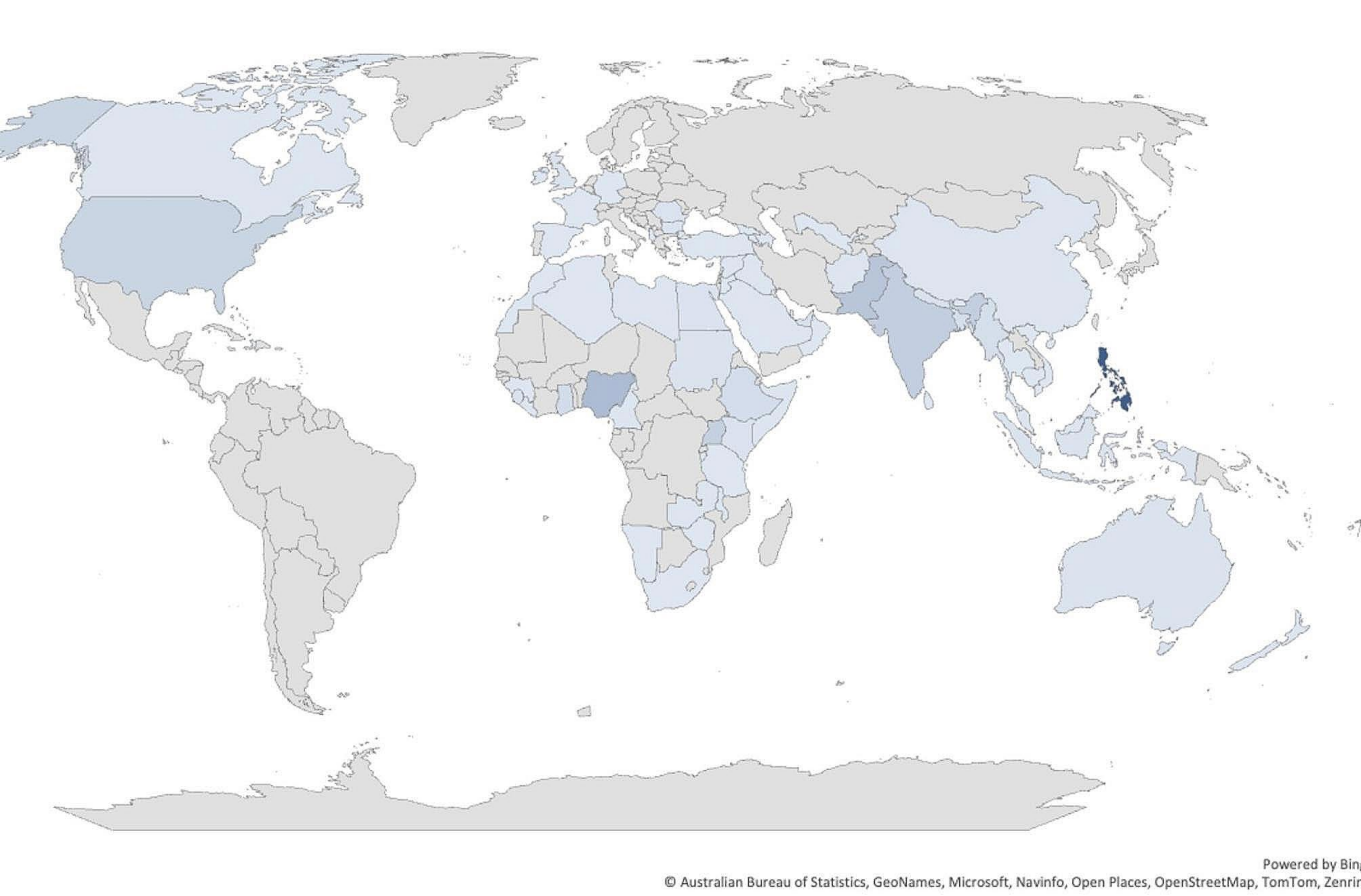
Self-reported country location of individuals completing the registry and hepatitis B discrimination experience (*N* = 569)

A total of 569 individuals responded to the survey between May 1, 2021, and December 31, 2023. Of these, 59% (*N* = 334) were male with the majority aged between 30 and 39 years (49%; *N* = 277, followed by less than 29 years (32%; *N* = 182), 40–49 years (15%; *N* = 85), 50–59 years (3%, *N* = 16) and more than 60 years (2%; *N* = 9). Individuals identified as residing in the Philippines (34%; N = 194), Nigeria (11%; N = 60), Pakistan (8%; N = 45), India (6%, N = 34), Uganda (5%; N = 31), the United States of America (4%, N = 26), Ghana (3%; N = 15), Ethiopia (2%; N = 14), and other countries in smaller number with a total of 65 countries reported discrimination at least by one individual; Fig. 1).

A total of 87% (*N* = 491) of the respondents had personally experienced discrimination because of their hepatitis B status, and 58% (*N* = 327) knew of someone living with hepatitis B who has experienced hepatitis B-related discrimination (Table 1). Most responses indicated discrimination took place by an employer (43%, *N* = 234) when applying for a travel or immigration visa (27%, *N* = 147), within a health facility or hospital (19%, *N* = 102), within an educational setting (14%, *N* = 76), or in a private home or residence (10%, *N* = 54) (Table 2).

**Table 1:** Demographic characteristics of individuals completing the discrimination registry for hepatitis B

Question	Sample response (*N*)	Percentage
**Age**		
0–17 years	3	0.53%
18–29 years	179	31%
30–39 years	277	49%
40–49 years	85	15%
50–59 years	16	3%
> 60 years	9	2%
**Gender**		
Male	334	59%
Female	277	40%
Prefer not to answer	8	2%
**Experienced discrimination personally**		
Yes	491	87%
No	76	13%
**Knows someone with hepatitis B who experienced discrimination**		
Yes	327	58%
No	236	42%

**Table 2:** The type of discrimination corresponds with the top eight countries in which discrimination was reported to the registry *

Country	Discrimination type employer	Online	Public	Health facility	Private residence	Education setting	Place of worship	Travel or work visa	Relationship
Ethiopia (*N* = 19)	8	0	0	1	3	2	1	4	0
Ghana (*N* = 15)	5	0	0	2	1	2	0	4	1
India (*N* = 47)	11	1	3	4	6	2	1	15	2
Nigeria (*N* = 70)	22	3	4	13	4	8	0	6	3
Pakistan (*N* = 67)	24	1	0	7	3	4	0	24	1
Philippines (*N* = 284)	97	9	13	25	18	21	7	87	0
Uganda (*N* = 48)	23	0	2	8	4	8	2	43	0
United States of America (*N* = 42)	14	4	3	13	3	2	0	1	0
Total	138	13	16	47	35	31	10	80	6

*This table displays multiple responses from individuals from the highest sampled countries

### Qualitative findings

A total of 461 individuals filled out the open-ended section of the survey to provide additional detail on their experiences of discrimination. Most related to limits on work visas, followed by in-country employment restrictions related to hepatitis B, educational-based discrimination, discrimination within the community and health facilities, and the emotional impact of hepatitis B discrimination. Each of the experiences is presented as themes with corresponding verbatim quotes in the following subsections.

### Work visa discrimination

Most people participating in the survey and who completed the open-ended responses reported discrimination when applying to work in overseas countries. Of these individuals, most of whom were from the Philippines, Nigeria, Ghana, or Uganda, the majority reported being denied work permit in the Gulf Cooperation Council countries including Bahrain, Kuwait, Oman, Qatar, Saudi Arabia and the United Arab Emirates to support their families in their home country. This is reflected by an individual from the Philippines who shared, “*When I am applying abroad as a Domestic Helper bound [for] Kuwait [I] found out that my hepatitis B (HBsAg) is reactive and told me that I am unfit to work there.”* Similarly, another individual reported, *“[In] 2022 I got rejected in residence permit by Qatar Government even though I already got employment by the company.”* Another individual from Kenya described facing deportation because of hepatitis B and stated, *“It happened in the state of Qatar, I had just gone to work as a cleaner and it was mandatory for everyone to get screened, I got screened and I was highly discriminated, I was given 3 days to leave Qatar or face deportation.”*

Despite reporting as being healthy and at low risk for transmission or serious liver disease, participants reported that this health status did not support visa approval, and individual from India shared,*“When I got [a] job in Bahrain, as part of medical checkup they denied me since I [am] hepatitis B positive. I mentioned that viral load is minimal and [I am] not taking any medicine.”* Another individual from the Philippines shared that while their doctor approved their work and travel, their request for visa was rejected: *“When I applied [for] that agency for Japan when they know the result that I have hepatitis B they automatically unfit me to work and reject my application even though my doctor told me I am fit to work.”*

The impact of not having work visas approved not only affected individuals with hepatitis B, but also their families in their country of origin with one person from the Philippines noting, *“I tried several times to work abroad to help my parents and my son, but every time I tried it was always not possible because of hep[atitis] B.”*

### In-country employment discrimination

While applying to work in other countries was a major source of discrimination, employment-related discrimination in home countries was also a significant contributor to discrimination experienced by those living with hepatitis B.

In most endemic countries, chronic hepatitis B infection primarily occurs with the virus being transmitted from mother to child in and around birth. Many respondents’ discrimination occurred in contexts in which the risk of hepatitis B transmission is negligible, including within office environments. One person from the Philippines explained, “*I was applying [for] a job for the position of office staff and they required a series of test[s]. They found that I’m hepatitis B reactive but the doctor said … I’m physically fit and healthy, I don’t have the symptoms and the employer denied my application …. It’s hard for me to find work I’m unemployed because of this virus*.” Another individual similarly shared, “*In Uganda, I couldn’t be given a job in a manufacturing company because of my (hepatitis B) status*.”

Other respondents described the economic challenges associated with this discrimination. An individual from the Philippines shared, *“[In] 2014 I stopped my work because my employer know that I tested hepatitis B positive. I felt depressed that moment because I’m a breadwinner of the family and lost my job because of my illness even [though] I’m fit to work*.”

#### Military discrimination

Several individuals reported discrimination taking place in the Philippines, the United States, Uganda, and Nigeria when applying to serve within their police force or military. One individual shared, “*People think this is a sin to be HBV positive. Also, I can’t join [the] military for it. Also, I’m unfit for some government job in my country for it*.” Similarly, another from Nigeria shared, “*I wanted to be a military man. After scaling through all the rigorous exercise and screening processes, I was shown the exit gate because of my hepatitis B status. Even though my test result showed that the virus is inactive, no one wanted to pay attention to my plea. I felt bad and cheated*.” Another shared, “*I joined the US Navy and started my training. After 6 weeks of training the medical office called me one day and told me that I couldn’t finish my training because they found hepatitis B in my blood and the Navy said they cannot keep me*.”

### Educational discrimination

Other individuals experienced discrimination when applying for educational opportunities both within their own countries and for international study. One individual from Sudan shared, “*Me and my family were diagnosed as hepatitis B positive. The school was notified that me and my brothers were positive. The principal of the school kicked us out of the school*.” Another respondent from Pakistan shared, “*I applied for abroad studies but reject me because my HBsAg is positive. I am a gold medalist in the medical field but now I can’t do any work or study next due to this. Now I am thinking that I waste my study and [at] times feeling very unlucky man in this world.*”

Other respondents highlighted the policies in place in certain countries and institutions that prevent people with hepatitis B from studying and an individual from Nigeria shared, “*I got Ph.D. sponsorship to study at University Putra Malaysia, I was not notified when I was filling the application form that the institution prohibits hepatitis B carriers from acquiring education there*.” Others shared they are treated differently within academic institutions because of their hepatitis B, with a person from Uganda noting, “*It happened at my university when I was in a lecture room and one of our facilitators said that people with hep B should not associate with the rest nor shake hands that the virus is more risky than any other virus*.”

### Interpersonal relationships/family discrimination

Respondents described being treated differently within the community by friends, family, and within relationships because of hepatitis B. One individual from the Philippines described immigration policy preventing their relationship progression and shared, “*I was denied a fiancé visa to Belgium because the state members considered me as a threat to public health*.”

Others describe hepatitis B being used against them in relationship conflicts as an individual from Nigeria described, “*The discrimination was by my husband, he decided to use my HBV as a claim for custody of our 2 children in [state court removed for anonymity]. Also claimed I couldn’t get a job due to HBV in our residence in Abu Dhabi but he was the one that refused me to work. I just need help in order to get my children, since they are vaccinated at birth*.” Similar situations were described in Sudan, Ethiopia, Sierra Leone, and India regarding marital disputes and hepatitis B.

Others described premarital testing for hepatitis B from India and described, “*Marriage rejection due to hepatitis B, so need a separate marriage group for hepatitis B people*.” Another individual from Morocco described being treated differently at home because of hepatitis B and explained, “*In my country between family my own mother said to others brothers and sisters that everyone should be careful with me because I have hepatitis B. Mother said from now I would not eat with, share the same spoon or plates and sleep no one in this family I became shame to myself*.”

### Health facility discrimination

Respondents reported the discrimination experienced within health facilities as patients and as healthcare workers.

A woman in Liberia described the impact of issues related to poor confidentiality within health systems, “*I got pregnant when (in) another hospital I was again diagnosed of having the virus…but before I was told by a nurse about the condition almost everyone in the hospital knows my condition people started to look at me in a guilty manner…the second time I entered the hospital and this time I only when to do my ultrasound ….and I broke down thinking even at birth if they would pay attention to me .they never wanted to touch me and even do my vitals sign to know my pressure*.”

Poor knowledge about hepatitis B among health professionals provided the context for very poor health care. An individual in Nigeria described, “*A medical doctor told me not to bother myself with antiviral drugs or livolyn forte (a Thailand based liver tonic). That it’s just a waste of money and time, that I will die with this simple. I was expecting him to give me some assurance of beating the HBV given the fact that I don’t have the symptoms even after I have always tested positive since 2011*.”

It was not only as patients that health facility-related discrimination occurred with one nurse sharing challenges with employment because of hepatitis B and stated, “*Chicago, Illinois, U.S.A. I work as a registered nurse. I have to take a hepatitis B blood test yearly, so I tested positive. My manager told me to gives him a resignation paper. This is because he believes I can give hepatitis B to my patients if I work in dialysis and deal with blood*.”

### The emotional impact of hepatitis B discrimination

Discrimination was reported to significantly impact the lives of people with hepatitis B. Individuals spoke of the multi-faceted impact of discrimination on their mental health and the lives of people with hepatitis B. An individual shared, “*My world fell apart that time and experienced depression. Being a nurse is a childhood dream of mine and it was taken away from me because I have hep B. I lost my friends and my girlfriend dumped me upon knowing my status. It was tragic experiencing that. I lost my purpose and my dream*.” One individual from Tanzania described the impact of discrimination overall and shared, *“[The] consequence of this stigma and discrimination is social isolation and affects our mental health and slow[s] down and academic performance as most of time we think about hepatitis and how we going to be treated while our economic situation is very difficult*.”

Many respondents emphasized the emotional toll that hepatitis B discrimination has on their lives from “feeling down,” depressed, hopeless, and an overall impact on mental health and psychological well-being. In open-ended responses, people noted feeling unlucky or suicidal: “*I waste my study and time feeling very unlucky”*, “*I was so disappointed till to date that I even think of losing my life because it is so hurtful*.”

## Conclusion

This is the largest primary collection of hepatitis B-associated discrimination events to date. Our results show that hepatitis B-related discrimination is a global issue, occurring in broad settings: employment settings (both domestic and for international work visa opportunities), academic and educational prospects, at the community level, within health care settings, and within relationships for premarital screening or domestic partnership disputes. Within many countries including Saudi Arabia and Pakistan, premarital screening for hepatitis B frequently takes place and individuals who test positive are often not allowed to marry and face discrimination from families that decide not to move forward with the relationship. Further research is needed to continue to highlight the burden, breadth, and impact of premarital screening for hepatitis B as much has only been reported anecdotally. Additionally, the use of hepatitis B within relationship disputes should continue to be explored further as it was described within the registry data.

While most individuals are healthy, able, and motivated to work to support their families, hepatitis B discrimination clearly plays a significant role in the lives of individuals and their families. Often, individuals were forced to be tested, were tested without their knowledge, or were not asked permission before being tested. Discrimination was reported to occur at multiple points throughout the lives of participants as individuals were denied their rights to work, support families, and accomplish their goals of happiness and career choices. Several individuals shared experiencing discrimination when applying for employment, seeking educational opportunities, and essentially affecting their personal relationships. This further clarifies the significant impact that hepatitis B discrimination has on people’s lives, and for some others cyclically focusing individuals to relive trauma and rejection [5]. Ultimately, this discrimination can have a negative impact on health outcomes for those with hepatitis B including the possible avoidance of health care services, routine management, mental and emotional health, and social isolation as it has been reported in other disease states [17, 18].

Hepatitis B discrimination in part can be caused by the overall lack of knowledge associated with hepatitis B in society. Instances of misunderstanding over infection transmission and disease progression were prevalent in the reports. These can translate into unjust and consequential uncertainty and fear of the disease [9, 19, 20]. From our data, it is clear there is a knowledge gap even among health providers in terms of hepatitis B transmission and clinical management. This knowledge gap has been related to hepatitis B within the literature and has a significant impact on stigma and discrimination globally [19, 21–23]. Education both at the provider and community level in mass form should be prioritized to ensure accurate information about hepatitis B disease is disseminated globally. Supportive services should also be implemented for people living with hepatitis B to help cope with the unjust discrimination experienced.

The hepatitis B surface antigen unfortunately and unjustly is used as a tool for discrimination which limits opportunities for people with hepatitis B. Public health tools such as vaccination and antiviral medication reduce viral load, which makes education, employment, international work, or travel opportunities safe regardless of hepatitis B. Some countries do have policies in place to prevent discrimination from occurring. For example, the United States within its civilian population has protections to prevent discrimination under the Americans with Disabilities Act (ADA); but discrimination still occurs within U.S. military settings and policies do not always disseminate into practice [24]. Australia also has a similar policy in place to prevent discrimination against people living with hepatitis B [25]. China also has a policy to prevent discrimination, but it is also unclear the extent to which this policy is actually followed by employers on the ground [10]. With the need for robust policies and protections to prevent discrimination, there is a need to also ensure that protective policies are widely disseminated and enforced at the local levels so that discrimination is not occurring.

An important area where future research is needed is exploring the economic impact of hepatitis B-related discrimination. Our data demonstrate that hepatitis B clearly impacts one’s ability to obtain work, ultimately limiting one’s economic opportunities. Limiting a person’s economic opportunities could also inhibit access to treatment and health care as funds are required to access both resources in most settings. For people experiencing discrimination priorities are focused on finding employment and economic stability rather than treatment and/or management related to hepatitis B thus further perpetuating the consequences of discrimination. Our research identifies the emotional and social implications of hepatitis B discrimination for people with lived experience, but future efforts should explore the true economic burden of discrimination.

As we work towards the World Health Organization Hepatitis B Elimination goals which call for increased testing, we should also ensure that there are protections in place to prevent this discrimination as reported by so many. We need to question the ethics of scaling up global testing for hepatitis B without adequately protecting people who test positive for hepatitis B infection and should work with governments to ensure protective policies are implemented. Additionally, supportive services like peer navigators and educators, and mental health resources should be resourced to help individuals manage hepatitis B and reduce some of the emotional burdens faced by those with hepatitis B.

## Limitations

Our registry is not representative of all the lived experiences associated with hepatitis B discrimination and has limitations. First, the survey was only available in English language only on the Survey Monkey platform, limiting its generalizability. While we think we have a large sample size and reached saturation of data, there are major global gaps of note. From previously published studies on hepatitis B discrimination in China and Vietnam, we would expect to have more responses from these areas [12, 26]. However, we had low representation from these countries, this is likely due to the ability to access the survey platform in China and the fact that our survey was only available in the English language. There was large representation from the Philippines within our sample which reflects the high prevalence of hepatitis B in the country and discrimination policy within the Philippines [1]. This policy does not protect individuals with hepatitis B against discrimination but in many situations including immigration and employment discrimination openly occurs legally [27]. Additionally, our survey captures data mostly from middle-aged adults, it is important to have more representation from other age groups, particularly elder adults, youth, and adolescents related to discrimination. More research is needed to fully understand the lived experience of discrimination related to hepatitis B globally.

### Contributions to the literature

- This is the largest primary collection of hepatitis B-associated discrimination events to date from individuals with hepatitis B.
- Our results show that hepatitis B-related discrimination is a global issue, occurring in broad settings including employment settings (both domestic and for international work visa opportunities), academic and educational prospects, at the community level, within health care settings, and within relationships for premarital screening or domestic partnership disputes.
- These findings contribute to the understanding of the negative and broad impact hepatitis B discrimination has on people living with it.

## Data Availability

De-identified survey data is available upon reasonable request by contacting the corresponding author at Catherine.Freeland@hepb.org.
